# A Facile Method to Incorporate Di‐Dopant Elements (F and Sb) into Crystalline Mesoporous Tin Dioxide Nano Powder at Ambient Temperature and Pressure

**DOI:** 10.1002/open.202400096

**Published:** 2024-11-16

**Authors:** Tariq Aqeel, Heather F. Greer

**Affiliations:** ^1^ Department of Science College of basic education The Public Authority for Applied Education and Training (PAAET) P.O. Box 23167 Safat 13092 Kuwait; ^2^ Yusuf Hamied Department of Chemistry University of Cambridge Cambridge CB2 1EW UK

**Keywords:** Mesoporous, Tin dioxide, Fluoride, Antimony, Optoelectronic conductance

## Abstract

A simple two step synthetic method for di‐doped crystalline mesoporous tin dioxide powder containing antimony and fluoride at ambient pressure and temperature has been developed. This approach produced materials with high surface areas and improved electrical and optoelectrical conductance. The two dopant elements; antimony and fluoride were introduced to tin dioxide by two approaches. Both approaches produced mesoporous tin dioxide with antimony and fluoride that are integrated in the framework. The structures of these materials are analyzed by powder X‐ray diffraction, N_2_ sorption analysis, transmission electron microscopy, energy dispersive X‐ray spectroscopy and X‐ray photoelectron spectroscopy. The conductance of the materials improved by factor of 13–34 compared to undoped mesoporous tin dioxide. The effect of the di‐doped elements on structure, conductance and optoelectronic properties of these materials are discussed in this paper.

## Graphical abstract

We successfully developed a reproducible and facile method to synthesize Fluoride– antimony doped mesoporous crystalline tin dioxide nanostructures under ambient pressure and temperature. This method consists of two steps introduction of elements without losing the main mesoporous structure of the material. The studies of the electrical and optical properties of the material indicate that we prepared a nanostructure with improved electrical conductance and attractive opto‐electronic properties.

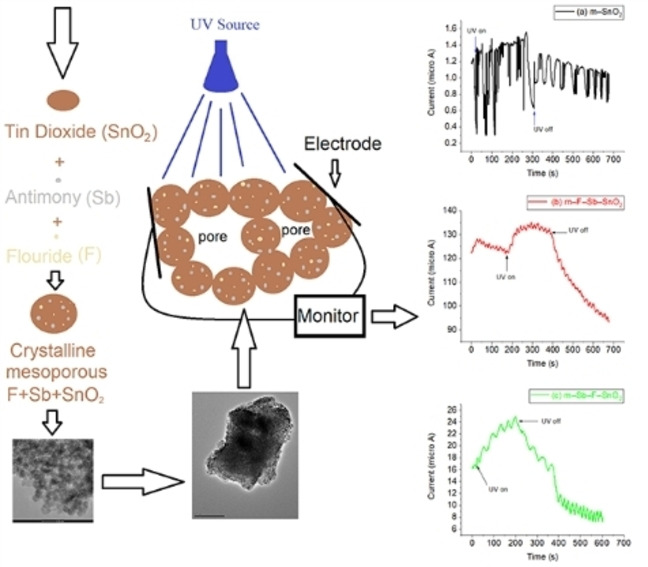

## Introduction

Recently, wide bandgap semiconductors have been extensively employed in opto‐catalysis and gas sensing devices. However, the low electrical conductance produced by these materials limits their implementation in portable devices. Therefore, introducing two different elements to the structure of nanosized metal oxide semiconductors could be a constructive approach to improve their electrical and opto‐electrical conductance. In this work, we aim to improve the conductance and optical conductance of mesoporous tin dioxide (m–SnO_2_). Tin dioxide is an n‐type semiconductor with a bandgap of 3.6 eV. It has been used extensively in many applications, for example in gas sensing,[^1^, ^2^, ^3^] optical catalysis and optical devices,[^4^, ^5^, ^6^] mostly due to its low costs, operational stability, rapid charge transfer and wide range gas sensitivity. The surface area, surface chemistry and crystal size play an important role in SnO_2_ performance. Altering SnO_2_ surface chemistry or morphology will affect the bandgap electrical and optical conductance.[^7^, ^8^] This can be achieved by different factors. 1) doping with different elements,[^8^, ^9^, ^10^] 2) introducing additional structural defects, 3) changing the crystal size[^11^, ^12^] and 4) creating pores in the structure.^[13]^

The introduction of foreign elements into the framework is known to improve electrical conductance, increase surface charges,[^14^, ^15^] increase defects, and sensing properties of m–SnO_2._ ^[16]^ Each element introduced, for example antimony (Sb^+x^) will enhance specific properties of SnO_2_.^[14]^ Antimony would replace the tin atoms in the framework.^[15]^ Additionally Sb possesses many oxidation states and each state will affect the main bandgap differently.[^9^, ^17^] By adding more electrons to the conducting bands in the case of Sb^+5^ or by making more structural defects such as oxygen vacancies, in the case of Sb^+3^,[^9^, ^18^, ^19^] this would introduce an additional “defect band” (close to the band edges) above the covalent band Sb^+5^ and under the main conduction band Sb^+3^.[^17^, ^20^] This intermediate defect band decreases the main bandgap allowing more valance electrons to hop and conduct electricity at lower energies.[^21^, ^22^] Decreasing the bandgap is one of the key factors effecting implementations of semiconductor materials in gas sensing or optical detector devices.^[23]^ As a result, these materials will require less activation energy to react or conduct electricity. The original bandgap of tin dioxide is 3.6 eV[^21^, ^22^] and requires a high thermal energy of 300–500 °C to free electrons from the valance band to reach the conductance band. However, decreasing the bandgap, for example to ∼3 eV, will activate SnO_2_ at lower operating temperatures.^[21]^ On the other hand, replacing some of the framework oxygen with F^−^, will alter the bandgap by creating a defect band, an oxygen deficiency above the covalent band, which would also decrease the main bandgap of SnO_2_. In addition, F^−^ is an electron doner, which increases the overall number of charges and the conductance of SnO_2_.^[24]^ Moreover, Sb and F are more electronegative than Sn and O respectively. This extra electronegativity will strengthen/compact the framework which facilitates charge transfer, that contributes to the conductance increment of SnO_2_.

Moreover, introducing pores to a SnO_2_ structure increases the surface area significantly. It is expected that typical mesoporous tin dioxide (m–SnO_2_) has a surface area above 100 m^2^ g^−1^,[^13^, ^25^] whereas it is less than 10 m^2^ g^−1^ for a non‐porous structure.^[26]^ This dramatic increase in the surface area improves diffusion of analytes for catalysis or gas sensing and adsorption/desorption processes.[^27^, ^28^] It is found that both the size of the surface area and crystal size depend on the selected heat treatments employed during the calcination step of the synthesis.[^28^, ^29^] In addition, controlling the crystal size of SnO_2_ is essential towards tailoring surface sensitivity and charge flow (conductance).[^15^, ^30^] Creating a high surface area and decreasing the crystal size improves surface sensitivity towards low gas concentrations, surface redox reactions and the absorption/adsorption ability of SnO_2_ [^16^, ^31^] Moreover having a framework of connected crystals will improve the overall charge transfer in the material, which will influence the response of the material and conductance.[^13^, ^32^, ^33^] Therefore, in this work, we attempted to improve the conductance and optical conductance of m–SnO_2_ by introducing both Sb and F into its framework, controlling the crystal size, and crystal connectivity.

The synthesis of m–SnO_2_ crystalline nanopowders was performed by our developed precipitating method, which is based on a modification of the work of Severin et al.^[34]^ This facile modification produced m–SnO_2_ that endured multiple heat treatments up to 500 °C and it is highly reproducible.^[29]^ Therefore, we successfully employed this modified method in this project, to synthesize crystalline powders of m–F−Sb‐SnO_2_ and m–Sb–F–SnO_2,_ by two step procedures and two separate heat treatments. The analysis of the product's structure, morphology, chemical composition, electrical and optoelectrical properties will be discussed in this paper.

## Method and Material

### First Step

Mesoporous Sb–SnO_2_ or F–SnO_2_ was prepared by slowly stirring hexadecylamine (0.28 g) in ^i^PrOH (24.4 cm^3^) until completely dissolved. Next, Sn(O^i^Pr)_4_ (1.9 g) was added to the solution at room temperature, then SbCl_3_ (0.1 g) was added to prepare Sb‐SnO_2_ and H_3_NF (0.05 g) was added to prepare F–SnO_2_. The reactants were stirred slowly under water‐saturated air (approximately 80 % humidity) at atmospheric pressure for 3 days. The obtained product was filtered and washed with water and ethanol. The product was then transferred to a Soxhlet extractor and extracted with ethanol overnight (approximately 16 h), in order to remove the surfactant, and was subsequently collected by filtration. The product was then calcined at 300 °C in air for 1 h followed by 400 °C for 15 min (heating rate of 2 °C min^−1^) and subsequently labeled as m–Sb–SnO_2_ and m–F–SnO_2_.

### Second Step

Approach one: m–F–SnO_2_ (0.36 g) was heated at 150 °C for 2 h then mixed with SbCl_3_ (0.04 g) in ^i^PrOH (1.6 g) and mixed at 80 °C for 30 min. The product was collected by gravity filtration, dried at room temperature, calcined at 400 °C for 30 min (heating rate of 2 °C min^−1^) and then labeled as m–Sb–F–SnO_2_.

Approach two: m–Sb–SnO_2_ (0.82 g) was heated at 150 °C for 2 h then mixed with of NH_3_F (0.05 g) in deionized water at 40 °C for 30 min. The product was collected by gravity filtration, dried at room temperature, calcined at 400 °C for 30 min (heating rate of 2 °C min^−1^) and labeled as m–F−Sb‐SnO_2_.

The experimental masses should be Sb 6.0, F 2.1 wt.% in m–Sb–F–SnO_2_ sample and F 3.0, Sb 6.3 wt.% for m–F−Sb–SnO_2_.

### Characterization

Powder X‐ray diffraction (XRD) measurements were performed with a Bruker AXS D8 ADVANCE diffractometer using a copper target (*λ*=1.5418 Å). Analyses were carried out with DIFFRAC^plus^ software, and the operational parameters were set as 40 kV; 40 mA; 0.1 mm low‐angle front slit window size, 1.0 mm for wide‐angle scans; 0.5–1.0 mm between the deflection plate and the sample for low‐angle scans; and 0.5 steps⋅s^−1^ in continuous coupled two‐theta scan/theta scan mode. A Micrometrics Tristar analyzer was used for nitrogen gas adsorption–desorption analyses. A Thermo Scientific Talos F200X G2 operating at 200 kV was utilized for TEM analysis. TEM images were acquired using a Ceta 16 M CMOS camera. Scanning transmission electron microscopy (STEM) were collected using a Fischione high angle annular dark field (HAADF) detector at a camera length of 98 mm. To determine the sample chemical compositions energy dispersive X‐ray (EDX) spectra and maps were acquired using a Super‐X EDX system which consists of 4 windowless silicon drift detectors. The samples were prepared by pipetting 3 μL of an ethanol suspension onto a lacey carbon 300 mesh Cu grid. X‐ray photoelectron spectroscopy (XPS) data was acquired with a Thermo ESCALAB 250Xi spectrometer equipped with a monochromator and Al−Kα radiation source (1486.6 eV), and an Avantage data system was employed for spectral recording and processing. The XPS measurement parameters were set as: chamber pressure of <10^−9^ Torr, step size of 0.1 eV, dwell time of 100 ms, and a pass energy of 20 eV. The C 1s line (284.6 eV) of adventitious carbon was used to determine binding energy values. Neutralization of charge build up on the surface of the insulating layer in the standard charge compensation mode was achieved with a flood gun. For the UV tests, the samples were pressed into 10 mm diameter pellets. The UV opto electrical test was performed employing a UniEquip UV source of a Hg tube lamp (365 nm, 50–60 Hz, 24 W, and 230 V). 1.5 V was applied and the current was measured using an Agilent B2901 A source/measure unit and two banana probes placed 4 mm apart at room temperature.

## Results and Discussion

### XRD

**Figure 1:**
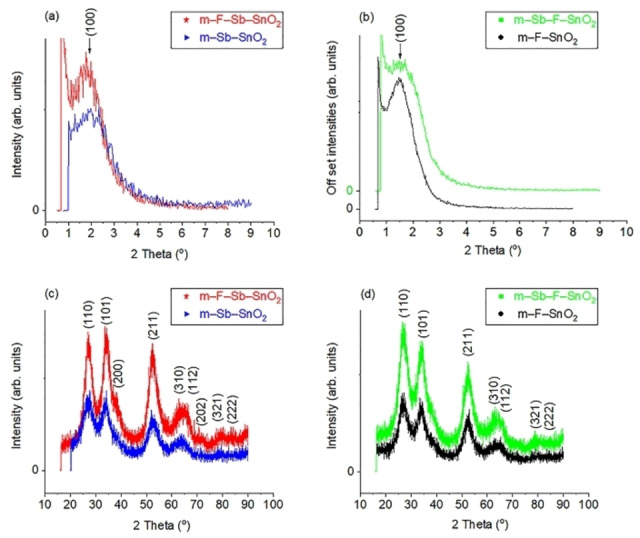
XRD patterns; (a) and (b) are the low angle scans, (c) and (d) are the wide angle scans for (▸) m–Sb–SnO_2_, (*) m–F−Sb–SnO_2_, (•) m–F–SnO_2_, (▪) m–Sb–F–SnO_2_

Low angle XRD patterns presented in Figure 1(a,b) reveal that the first step (introduction of a mono dopant to m–SnO_2_) of the synthesis was successful after the first calcination step and produced mesoporous m–Sb–SnO_2_ (Figure 1(a),▸) using approach one and m–F–SnO_2_ (Figure 1(b), •) using approach two. In addition, Figure 1(a,b) also confirms that the second step of the synthesis (introduction of the second dopant element into the mesoporous material) was also successful for both approaches after the second heat treatment. During this second step, approach one produced (*) m–F−Sb–SnO_2_ and approach two (▪) produced m–Sb–F–SnO_2_. The presence of the main diffraction (100) peak in all synthesized materials confirms that both approaches are successful. This main (100) peak shifted slightly to a lower 2Ɵ angle for sample (*) m–F−Sb–SnO_2_, indicating a larger *d*–spacing after the second heat treatment. This can occur from two factors: larger pores or walls. N_2_ analysis confirms that m–F−Sb–SnO_2_ sample has a slightly larger pore size. However, the absence of other peaks indicates that the pores do not possess long range order, mainly worm type pores. In addition, the shape of that main (100) peak did not change after the second step indicates that all the dopant elements are well incorporated in the framework of SnO_2_ and did not disrupt the pore structure.[^35^, ^36^]

The wide angle XRD scans presented in Figure 1(c,d) show that all materials are crystalline, having one phase of synthesized SnO_2_.^[19]^ The diffraction peaks are assigned to Cassiterite (tetragonal) structure of SnO_2_.[^21^, ^35^] No additional diffraction peaks appeared for Sb_x_O_y_ or a F species indicating no clustering outside the framework.[^37^, ^38^] However, amorphous phases cannot be ruled out. This also confirms that substitution of Sb and F ions did not affect the crystallinity of the material and Sb^[19]^ and F ions are inserted into the framework.^[24]^ Moreover, the wide angle XRD patterns after the second step had higher peak intensities for both approaches, which indicates an increase in the crystallinity of both materials,^[38]^ as a result of the second heat treatment.^[37]^ In addition, the (101) diffraction peak has a higher intensity than the (110) peak in sample m–F−Sb–SnO_2_ (Figure 1(c)) which is the opposite to that observed in sample m–Sb–F–SnO_2_ (Figure 1(d)). This indicates that the F concentration is larger^[38]^ in sample m–F−Sb–SnO_2_ compared to m–Sb–F–SnO_2_, which is confirmed by XPS and EDX results, because F atoms favor the occupation of (101) plane. This also indicates that F^−^ is readily incorporated in the framework.[^10^, ^24^]

### N_2_ Analysis

**Figure 2:**
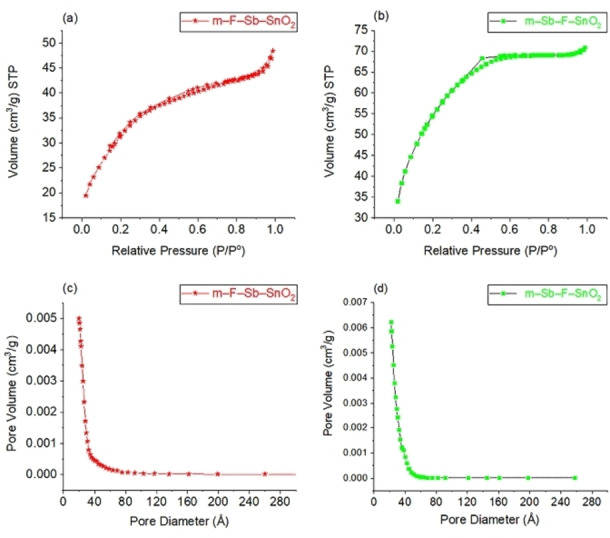
N_2_ sorption analysis for the final products after the second step of the synthesis; (a) isotherm, (c) pore size distribution for (*) m–F−Sb–SnO_2_ and (b) the isotherm and (d) pore size distribution for (▪) m–Sb–F–SnO_2_.

The N_2_ sorption analysis (Fig. 2) was performed for materials (*) m–F−Sb–SnO_2_ and (▪) m–Sb–F–SnO_2_ after the second (final) step of the synthesis for both approaches. Both samples produced similar isotherm curves as presented in Figure 2(a,b). These isotherms are of IVa type, having a H4 hysteresis loop, typically produced by mesoporous materials, according to IUPAC classification.^[39]^ Moreover, hysteresis loops are present in both isotherms as a result of the difference between the gas adsorption and desorption rates. Rate difference occurs due to the slower desorption rate caused by capillary condensation of the gas phase below saturation pressure *P* ^*o*^ into some pores that reached critical size, of 4 nm or larger.^[40]^ Furthermore, the hysteresis loop of sample m–F−Sb–SnO_2_ extended from a relative pressure range of 0.8 – 0.2, larger than that of m–Sb–F–SnO_2_ which is 0.5 – 0.3. This indicates that m–F−Sb–SnO_2_ has a high quantity of pores larger than 4 nm compared to m–F−Sb–SnO_2_. Moreover, Figure 2(c,d) confirms that both materials contain pore sizes of 4 nm or larger. However, Figure 2(c,d) shows the pore volume absorbed is greater for m–Sb–F–SnO_2_ than that of m–F−Sb–SnO_2_. This indicates that m–Sb–F–SnO_2_ has smaller pore sizes than m–F−Sb–SnO_2_. This is also confirmed by the BJH results for both samples. The average pore diameters are 2.6 and 2.3 nm for m–F−Sb–SnO_2_ and m–Sb–F–SnO_2_, respectively. In addition, the amount of N_2_ adsorbed by sample m–Sb–F–SnO_2_, shown by the y‐axis in Figure 2(b), is greater than that of sample m–F−Sb–SnO_2_ Figure 2(a), indicates that the surface area of m–Sb–F–SnO_2_ is higher. This is also confirmed by the BET surface areas results, which are 114 m^2^g^−1^ for m–F−Sb–SnO_2_ and 195 m^2^g^−1^ for m–Sb–F–SnO_2_, respectively. These values confirm that having smaller pores in the material increases the surface area.

### XPS

**Figure 3:**
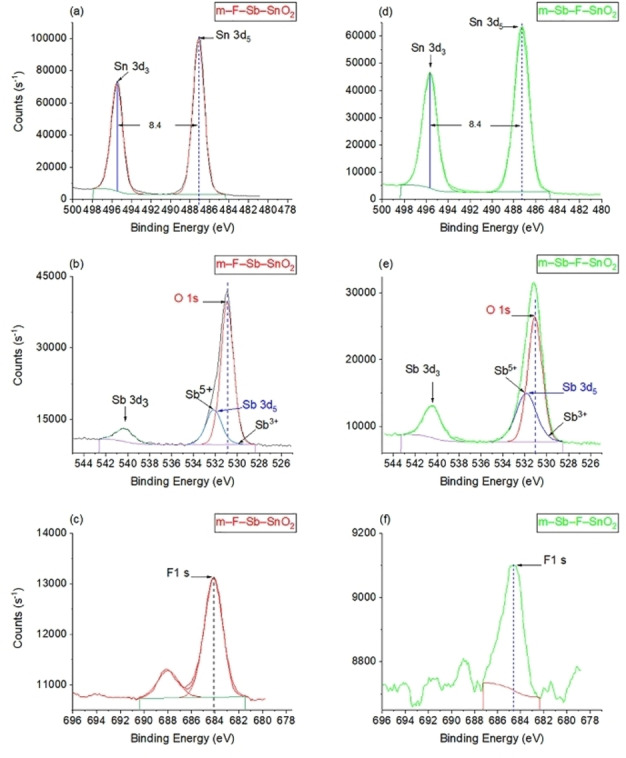
XPS results for (a, b and c) m–F−Sb–SnO_2_, (d, e and f) m–Sb–F–SnO_2_.

**Figure 4:**
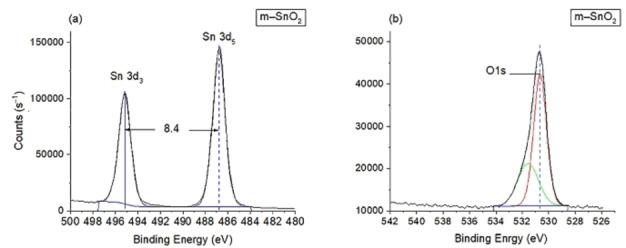
XPS results for pristine m–SnO_2_, (a) Sn 3d and (b) O1s

XPS analyses were performed on m–F−Sb–SnO_2_ and m–Sb–F–SnO_2_ to investigate their oxidation state and the surface/slight subsurface constitutes (Figure 3). Both materials had similar XPS results (Figures 3(a) and (d)) as follows: the binding energies of Sn, spin coupling of the 3d orbit for peaks at 3d_5_ at 487.1 eV and 3d_3_ at 495.5 eV having^[41]^ an orbital splitting of 8.4 eV,^[19]^ which are related to Sn^4+^ oxidation state of SnO_2_ material.^[19]^ Moreover, the shift of Sn 3d_5_ from 486.7 eV of pristine SnO_2_ ^[38]^ (Figure 4a) to a higher binding energy 487.1 eV is caused by Sb doping^[41]^ and also could indicate the existence of a direct Sn−F bond.^[10]^ The XPS spectra in Figure 3 (b) and (e) represents the O and Sb peaks of m–F−Sb–SnO_2_ and m–Sb–F–SnO_2_, respectively. The Sb 5d_3_ peak is masked by the O 1s peak but these two peaks are resolved by deconvolution of the asymmetric peak into two distinguished peaks. First the O 1s spectra at 530.8±0.3 eV corresponding to the O^2−^ species of SnO_2_ framework.^[38]^ This peak has shifted from 530.6 eV for pristine m–SnO_2_ (Figure 4(b)) to 530.9 eV for m–F−Sb–SnO_2_ and to 531.1 eV for m–Sb–F–SnO_2_ as a result of increasing the Sb concentration in the samples.[^42^, ^43^]

Moreover, the second peak is Sb 3d_5_ at 531.8 eV is assigned to Sb^5+^, and the shoulder of that peak extending to 530.2 eV (Figure 3(b)) corresponds to the existence of Sb^3+^ state.^[42]^ This shoulder extended more towards a Sb^3+^ oxidation state to 528.9 eV for sample m–Sb–F–SnO_2_ (Figure 3(e)) than in m–F−Sb–SnO_2_. This indicates that m–Sb–F–SnO_2_ has more Sb^3+^ (Sb_2_O_3_) species than that in m–F−Sb–SnO_2_. However, at 540.5 eV the Sb 3d_3_ peak, which is well resolved, refers to Sb^5+^ oxidation of Sb_2_O_5_.[^19^, ^20^, ^43^] Moreover, comparing the sizes of 3d_3_ and 3d_5_ peaks of Sb in relation to that of Sn in each sample,^[40]^ shows that sample m–Sb–F–SnO_2_ contains 2.5 times higher Sb concentration than m–F−Sb–SnO_2_. In addition, both Sb peaks confirm the successful substitution of Sb moieties of Sn atoms in SnO_2_.^[44]^ This also supports the analysis of the wide angle XRD discussion that Sb was readily integrated in the framework of SnO_2_ and did not form any aggregates on the surface.^[44]^

The XPS results for F are presented in Figure 3(c) and (f) for m–F−Sb–SnO_2_ and m–Sb–F–SnO_2_, respectively. The binding energy at 684.5 eV±0.2 eV corresponds to F^−^ ion that replaced the oxygen (O^2−^) in the lattice of m–SnO_2_, becoming a single electron donor.[^10^, ^24^] Moreover, F wt.% calculated from XPS at.% data reveals that m–F−Sb–SnO_2_ contains 0.9 wt. %, whereas only 0.3 wt. % in m–Sb–F–SnO_2_. Both values are significantly lower than what was introduced in the synthesis procedure, which was ∼2.5 wt.%. Moreover, these amounts indicate that the introduction of F precursor in the second step yielded a higher quantity in the final product compared to when it was introduced in the first step. These values also indicate that only about 12–36 % of the introduced F is incorporated in the framework and the rest was washed out. On the other hand, Sb wt.% in the final products came very close to the calculated value during the post synthesis. Sample m–F−Sb–SnO_2_ has 2.6 wt.% Sb while sample m–Sb–F–SnO_2_ has 5.9 wt.% Sb. These values represent 41 wt.% when Sb introduced during the synthesis and 98 wt.%, when introduced in step two (as post treatment). This confirms that Sb can be readily introduced to the framework of m–SnO_2_ by any of the approaches during the synthesis (step one) or by post treatment (step two). Furthermore, both quantitative results of F and Sb gave higher wt.% yields of the dopants in the product, when introduced in post treatment of the material. We think that the massive decrease in dopants content occurred during the synthesis because of the continued washing for about 16 h in the Soxhlet extraction step. This step was applied to the as‐synthesized material before the calcination (heat treatment) step, which tends to strengthen the constituents’ bonds.

### TEM/EDX

**Figure 5:**
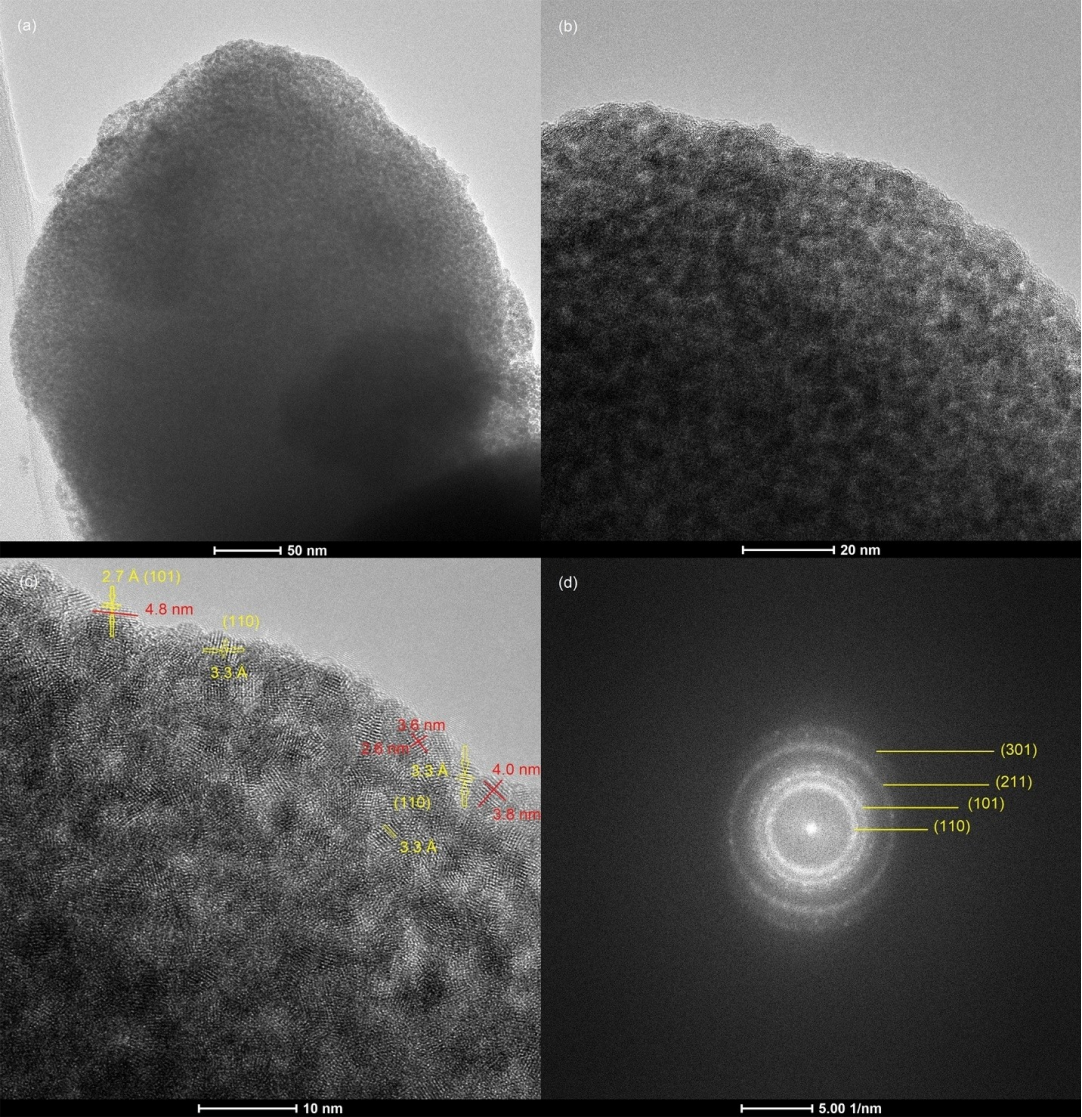
(a, b) TEM images, (c) HRTEM image of m–F−Sb–SnO_2_ and (d) is the corresponding FFT pattern of (c).

Figure 5 presents the TEM/HRTEM images of m–F−Sb–SnO_2_. Figure 5(a) is a low magnification image of the material, confirming that the whole particle is porous. Figure 5(b) is a selected area of image (a) showing that the material is chemically connected in a continuous form surrounding the pores. These pores are irregular in shape and size, as predicted by the low angle XRD. The size of the pores falls in the range of 2–4 nm. Image Figure 5(c) is a HRTEM image of a selected section of image Figure 5(b). This image reveals the crystalline nature of the material, a few of the lattice planes are indicated by the yellow arrows. These are the (110) 3.3 Å and (101) 2.7 Å.[^21^, ^25^]

This confirms that the introduction of Sb and F elements did not affect the crystallinity of SnO_2_ and was well incorporated in the framework. No aggregates of Sb or F were detected. Some of the crystal sizes are measured in image Figure 5(c) as indicated by the red lines. The average crystal size measured in image Figure 5(c) is 4 nm. Figure 5(d) is the corresponding fast fourier transform (FFT) pattern of Figure 5(c). It clearly shows the polycrystalline diffraction rings, (110), (101), (211) and (301) which are assigned to crystalline tetragonal SnO_2_, as indicated by the yellow lines in Figure 5(d). This supports and confirms the results obtained from the wide angle XRD.

**Figure 6:**
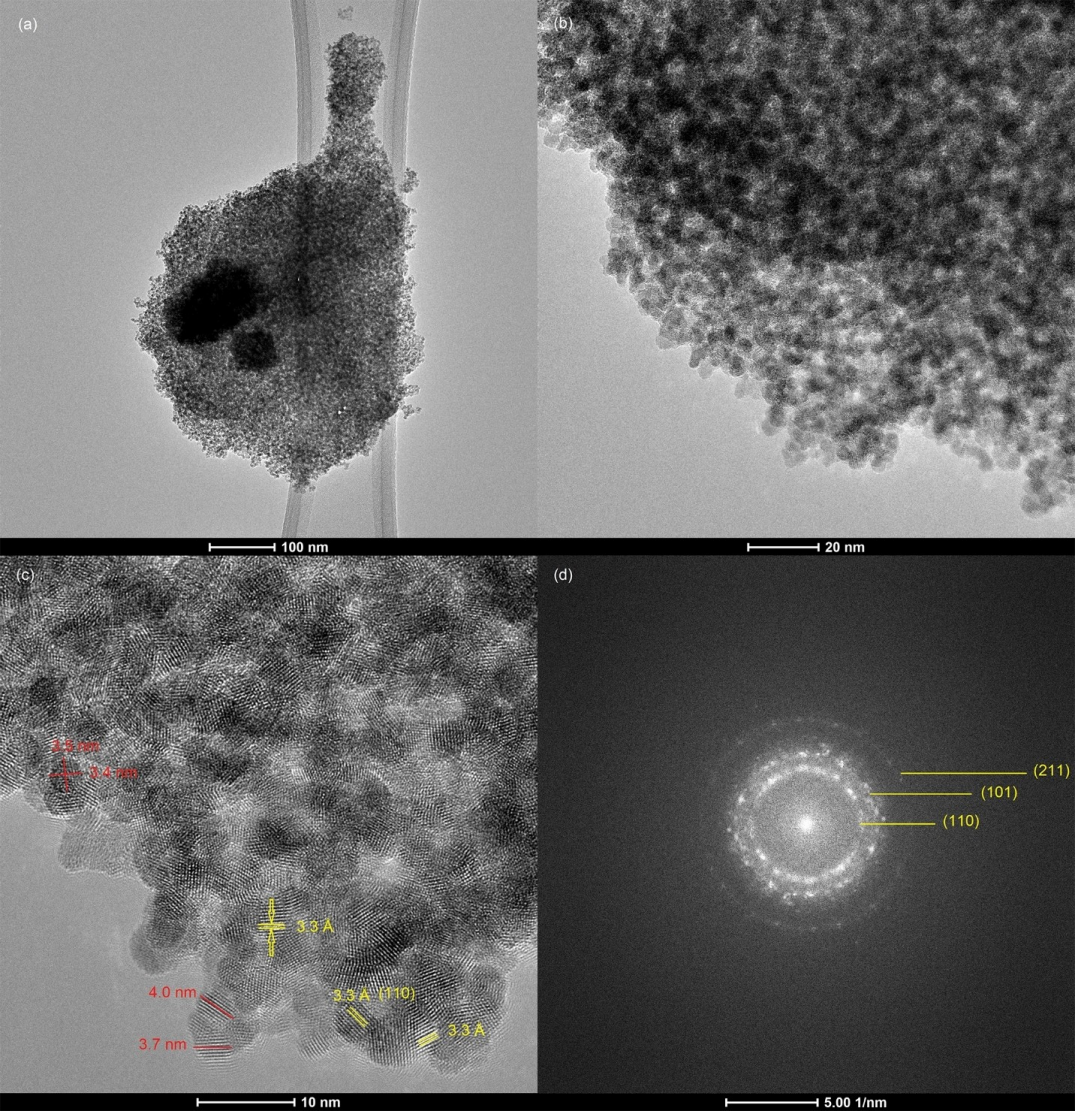
(a, b) TEM images, (c) HRTEM image of m–Sb–F–SnO_2_ and (d) is the corresponding FFT pattern of (c).

Figure 6 shows TEM images of m–Sb–F–SnO_2_. Image Figure 6(a) clearly shows that the entire material is highly porous. A higher magnification TEM image of the lower left region in Figure 6(a) is shown in Figure 6(b). This section clearly shows that the material is fused in continuous form surrounding the pores. The pores are not uniform in shape and size, as was detected by the low angle XRD data. The size of the pores falls between 2–3 nm. Figure 6(c) is a HRTEM image of a selected section of image Figure 6(b). This image reveals that the entire sample is crystalline, showing the crystalline planes, some of which are identified and indicated by the lines or arrows of one crystalline phase. These are the (110) 3.3 Å crystal planes of tetragonal rutile crystalline SnO_2_ structure,[^25^, ^38^] as indicated in image Figure 6(c), which also confirms that the introduction of Sb and F elements did not affect the crystallinity of SnO_2_ and did not produce a new phase. In addition, no aggregates of the dopant elements were detected. The average crystal size measured in image Figure 6(c) is 4 nm, some of which are indicated by the red lines, and they are more uniform with a spherical shape. Figure 6(d) represents the FFT pattern of this material. It clearly shows the diffraction rings, (110), (101) and (211), which are assigned to crystalline SnO_2_,[^25^, ^43^] as indicated by yellow lines in image Figure 6(d). This also matches the results obtained from the wide angle XRD.

**Figure 7:**
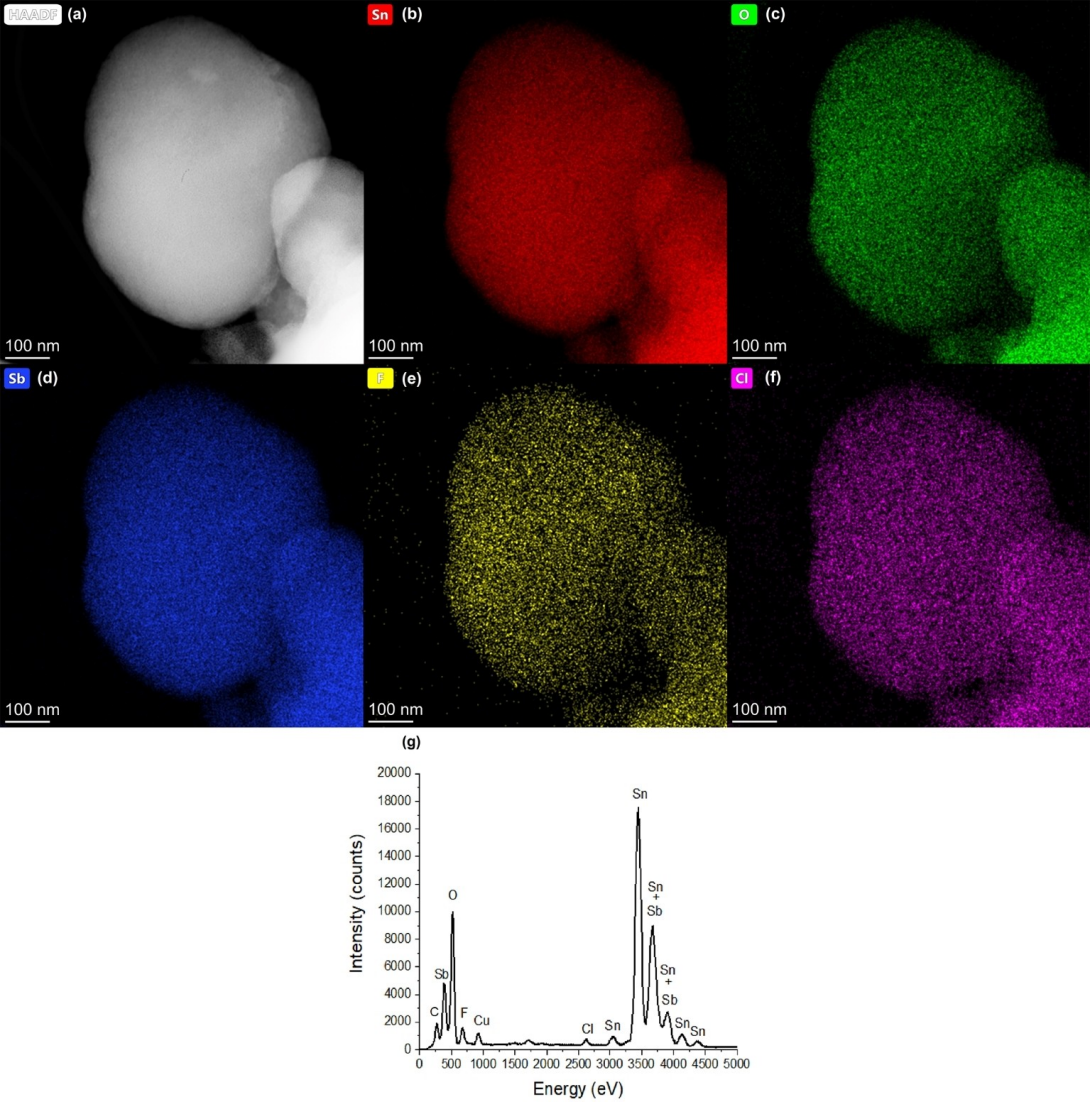
EDX elemental mapping (a) HAADF‐STEM image of m–F−Sb–SnO_2_ sample. EDX maps showing the distribution of (b) Sn, (c) O, (d) Sb, (e) F and (f) Cl. (g) The corresponding EDX spectra.

Figure 7 presents the EDX mapping of m–F−Sb–SnO_2_, (a) is the HAADF‐STEM image of a particle that was selected to show the elemental distribution of all elements in the sample. Figure 7(b) shows the distribution of Sn in this selected region of the sample. Figure (c–f) represents the elemental distribution of O, Sb, F and Cl. All elements are well distributed in the sample, no aggregates of Sb or F are detected. Figure 7(f) shows the existence of Cl in the sample, that might be adsorbed from Sb precursor (SbCl_3_) during the synthesis. The corresponding EDX spectra is presented in Figure 7(g), showing all K and L peaks of the elements that exist in the sample over a 0–5 keV. These elements are O, Sn, Sb, F and Cl. Cu is from the TEM grid which the sample is placed on during the analysis. The K_α_‐peak of F is detected at 0.67 keV, which confirms the existence of F in the SnO_2_. Quantification of the EDX spectrum suggests the F content is 1.1 wt.% and Sb content is 3.1 wt.%. These results are similar to that obtained from XPS results.

**Figure 8:**
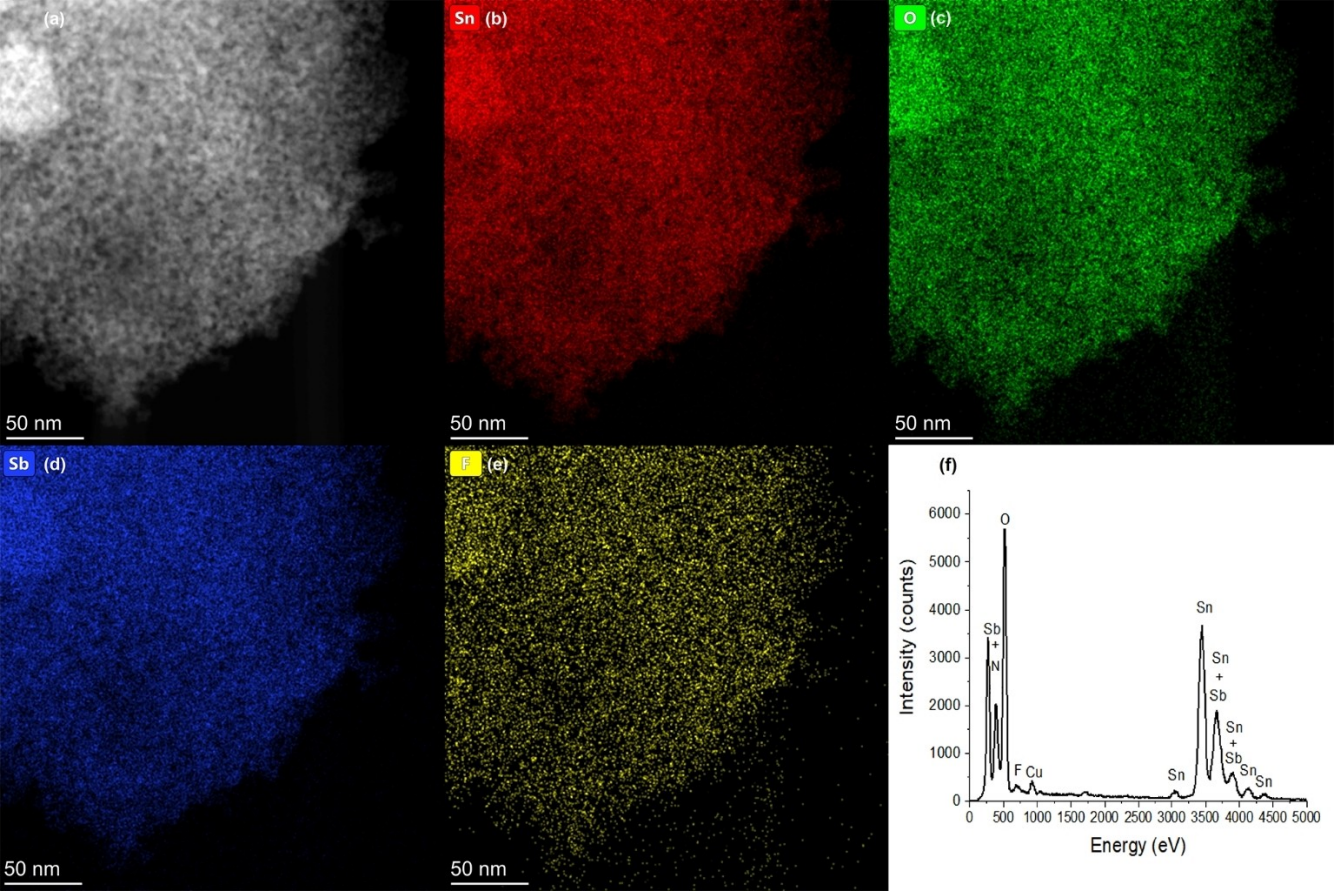
(a) HAADF‐STEM image, EDX maps of m–Sb–F–SnO_2_ showing the distribution of (b) Sn, (c) O, (d) Sb and (e) F. (f) The corresponding EDX spectra.

Figure 8 represents the EDX maps of the m–Sb–F–SnO_2_ sample. Image Figure 8(a) shows a HAADF‐STEM image of a particle, in which the pores appear darker than the material, and did not show any irregularities in the sample. The pores are well distributed in the entire sample. Figure 8(b) represents the distribution of Sn atoms in the same selected section of the sample whilst Figure 8(c–e) shows the O, Sb and F distribution. These atoms are well distributed throughout the materials and no other elements are detected. Figure 8(f) represents the EDX spectra of this section of m–Sb–F–SnO_2_, showing the K and L peaks of the constituents; Sn, O, Sb, F, and Cu, which is the grid composition. The quantitative analysis of this sample gave 5.9 wt.% of Sb and 0.5 wt.% of F in m–Sb–F–SnO_2_. These results are similar to that obtained from the XPS results. Furthermore, EDX and HRTEM did not show any aggregates of Sb or F species, which confirms uniform integration of these species in SnO_2_ framework, which support the wide angle XRD results.

## Conductance and Optoelectronic Test

### Conductance

The conductance of all samples was measured at room temperature (25 °C) in air and presented in Table 1. The conductance of the reference material, pristine m–SnO_2_ (prepared in a similar procedure) was the lowest, as expected. In sample m–Sb–F–SnO_2_ the conductance exceeded the reference conductance value by one order of magnitude. On the other hand, sample m–F−Sb–SnO_2_ gave the best conductance, which was higher than that of the reference material by two fold. This improvement of the conductance can result from a few factors. First, the doping effect at which the dopant elements and defects create an intermediate band that exists in the edges of the main valence and conductance bands of the SnO_2_. This intermediate band shortens the distance for the e^−^ to hop to the conductance band, which increases conductance of the material.[^42^, ^44^] The second factor is doping increases the number of charge carriers in the material, and more charges creates a higher conductance. In this case, both dopant elements are considered as an e^–^ doner species, Sb^+5[9,44]^ and F^−^,[^10^, ^24^] which increases the number of e^−^ in m–SnO_2_. The third factor is compacting the structure and decreasing the grain boundaries. F is more electronegative than O and Sb is more electronegative than Sn, this will contribute to compacting the framework structure of the final material, by shortening the chemical bonds, which facilitates the flow of charges. The TEM data in Figures 5 and 6 (images a, and b) confirm that m–F−Sb–SnO_2_ is more condensed than m–Sb–F–SnO_2_.

**Table 1:** Conductance of all samples at 25 °C.

Sample name	Conductance Ω^−1^ (S cm^−1^)
Undoped m–SnO_2_	5.0 * 10^−5^
m–F−Sb–SnO_2_	1.7 * 10^−3^
m–Sb–F–SnO_2_	6.5 * 10^−4^

Even though the quantity of Sb is more in sample m–Sb–F–SnO_2_ than in m–F−Sb–SnO_2_, the latter possesses higher conductance. This is because the existence of more Sb^+3^ moieties (in relation to Sb^+5^) in sample m–Sb–F–SnO_2_ than in m–F−Sb–SnO_2_, having more electron accepter Sb^+3^ species decreases the overall conductance of m–Sb–F–SnO_2_.^[9]^ Furthermore, XPS and EDX data confirmed that sample m–F−Sb–SnO_2_ has a higher F quantity than in m–Sb–F–SnO_2_, that also contributes to higher conductance of m–F−Sb–SnO_2_. Therefore, the conductance measurements agree with the quantities of dopant types and their oxidation states in the samples.

### The Optoelectronic Test

**Figure 9:**
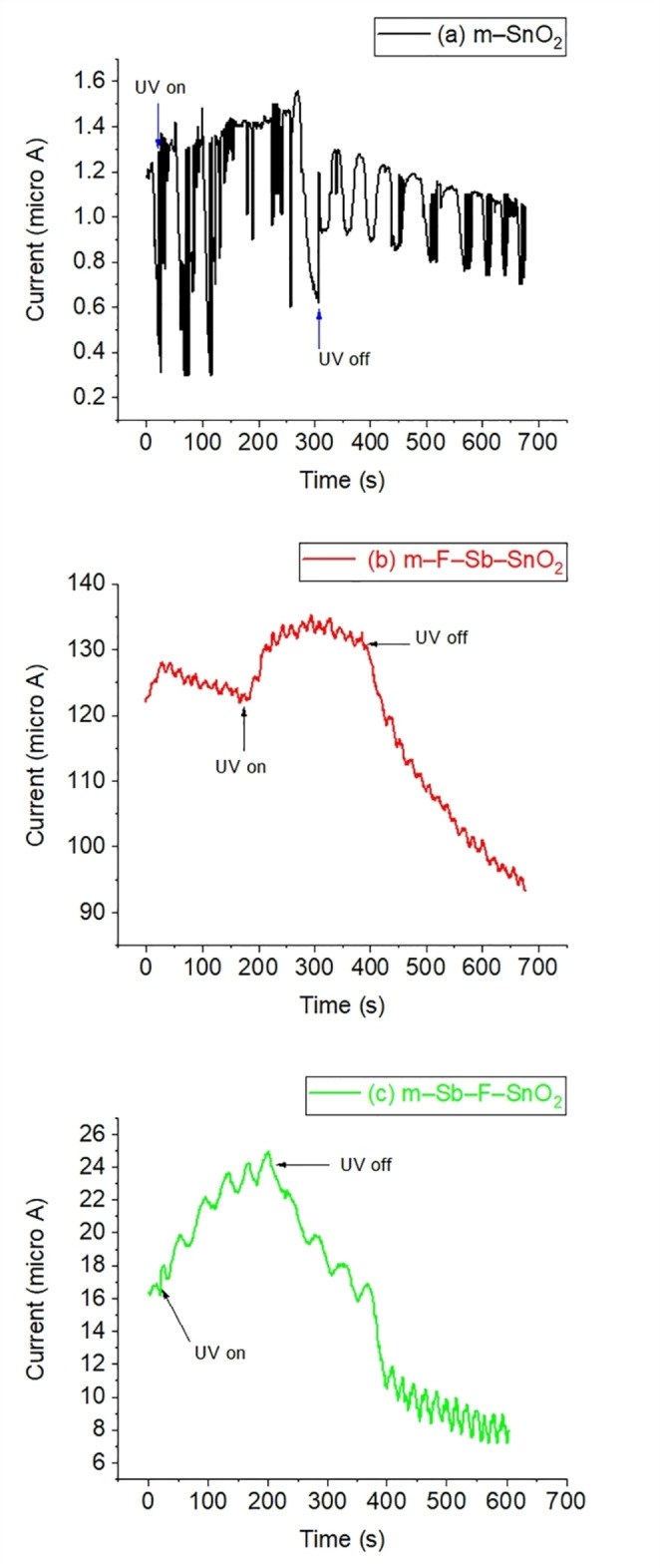
UV–irradiation effect on (a) m–SnO_2_, (b) m–F−Sb–SnO_2_, and (c) m–Sb–F–SnO_2_.

The materials optoelectronic properties were analysed by measuring the current produced by the materials during and after the irradiation of a UV source (Figure 9). Figure 9(a) represents the effect of the UV illumination of pristine m–SnO_2_. During this irradiation ∼3 min (excited state) the resultant current rose from 1.2 to 1.6 μA. When the irradiation was stopped (relaxed state) the current continued to decrease to ∼0.8 μA, eventually dropping below the initial state (at dark condition). We used pristine m–SnO_2_ as a reference material (as a base line of these materials) to determine the difference in current as a result of irradiation after doping. A similar effect was observed for both doped materials. Figure 9(b) presents the effect of UV–irradiation on the m–F−Sb–SnO_2_ sample. The material was stable (dark conditions) at ∼125 μA, then rose during the irradiation to 135 μA. After the irradiation is switched off the current decreased gradually reaching 95 μA at the end of the test (below the initial conditions). Figure 9(c) illiustrates the effect of the UV–irradiation test on m–Sb–F–SnO_2_ sample. The material was stablized at ∼16.5 μA, then rose during the irradiation to 25 μA. Then after the irradiation is stopped the current decreased gradually reaching 7 μA at the end of the test.

It is well documented that the semiconductors adsorb oxygen chemically (chemisorption) from air at room temperature.^[16]^ This adsorbed oxygen (molecular and dissociate (O_2_^−^, O^−^)) attached to the surface by attracting the surface electrons (e^−^), through an ionisation process creating a depletion layer, which decreases the semiconductor conductance. During irradiation the current increased as a result of the desorption of these ionic oxygens from the surface, at different rate, releasing these electrons back.^[44]^ This was observed by the initial increase in the current during the UV–irradiation of all of these materials. The second step occurred as a result of irradiation and is the movement of the charge carrier holes and e^−^ to the surface[^45^, ^46^] of the semiconducters and the UV energy facilitates the jump of the e^−^ to hop from the valence band to the conductance band of these semiconductors. This stepwise supply of electrons gave the gradual mountain shape peak observed on all the mterials tested in Figure 9. After that when the UV source is turned off, the excited e^−^ goes back to the covalent band and gradually the air oxygen re–adsorbed itself to the surface of the materials through (chemisorption) the attraction and binding with the surface e^−^, which decreases the current. The continuation of the current decrease below the initial starting point (the stabilization step) can be explained as follows. The structure of all these materials contains mesopores, which tend to absorb water molecules (hydrophilic nature)^[47]^ from the surrounding atmosphere if left exposed to air for a long period of time. This is why at the initial stabilization stage there are more water molecules physically absorbed in the materials, which gave the initial conductance reading. However, at the final stage of the test, there were less water molecules attached (compared to the initial stage) and more oxygen was chemically adsorbed decreasing the current to lower values than the initial one. Moreover, because both F^−^ and Sb^+5^ are electron doners species, the current produced by doped materials are higher by many folds than that of the pristine m–SnO_2_. The overall trend of this excitation current reflects similar trends as that of the relative conductance values of these materials, presented at Table 1. This also confirms that the difference in conductance between the doped materials is directly related to dopant quantities and their oxidation states in each sample.

Moreover, at this UV induced excited state, we believe these doped materials m–F−Sb–SnO_2_, and m–Sb–F–SnO_2_ can be readily employed as a gas sensor at room temperature. The sensor can be used without heating, especially at enviroment conditions at which gas concentrations exceed the lower explosive values. Typically the value of the bandgap energies of these materials are 3–4 eV, which require heating energy of 200 – 400 °C in order to be used as a gas sensor.^[31]^ This heating energy can be replaced by employing a UV source at room temperature. A few advantages of UV–induced gas sensing are: no heating elements required, preserving the materials constituents and oxidation states, maintaining the crystal sizes, increasing the charge carriers, and decreasing the intergrain boundaries.[^45^, ^46^]

## Conclusions

We have successfully synthesized crystalline mesoporous tin dioxide powder containing fluoride and antimony at ambient pressure and temperature (using our modified method). The crystals of these powders are chemically connected in a continuous framework. These materials m–F−Sb–SnO_2_ and m–Sb–F–SnO_2_ have a high BET surface area (114, 195 m^2^g^−1^) respectively, 4 nm average crystal sizes and a narrow pore size distribution of 2.5 nm. These materials possess high electrical conductance in air. The excellent improvement in conductance and optoelectronic conductance of m–F−Sb–SnO_2_ is attributed to higher concentration of F^−^ and Sb^+5^ moieties in the sample. These materials can be promoted to be employed readily as UV–induced gas sensors at room temperature.

## Conflict of Interests

The authors declare no conflict of interest.

1

## Data Availability

The data that support the findings of this study are available from the corresponding author upon reasonable request.
